# Bioactive Peptide Fractions from Collagen Hydrolysate of Common Carp Fish Byproduct: Antioxidant and Functional Properties

**DOI:** 10.3390/antiox11030509

**Published:** 2022-03-06

**Authors:** Diego J. González-Serrano, Milad Hadidi, Matin Varcheh, Aniseh Zarei Jelyani, Andres Moreno, Jose M. Lorenzo

**Affiliations:** 1 Department of Organic Chemistry, Faculty of Chemical Sciences and Technologies, University of Castilla-La Mancha, 13071 Ciudad Real, Spain; diegojesus.gonzalez@uclm.es; 2 Department of Chemistry, Faculty of Basic Sciences, Islamic Azad University, Arak Branch, Arak 96139-66549, Iran; m.varcheh@iau.ac.ir; 3 Food Control Laboratory, Department of Food and Drug, Shiraz University of Medical Science, Shiraz 71348-14336, Iran; a.z.jeylani@sumc.ac.ir; 4 Centro Tecnológico de la Carne de Galicia, Avd. Galicia Nº 4, Parque Tecnológico de Galicia, San Cibrao das Viñas, 32900 Ourense, Spain; 5 Área de Tecnología de los Alimentos, Facultad de Ciencias de Ourense, Universidade de Vigo, 32004 Ourense, Spain

**Keywords:** antioxidative activity, functional property, solubility, common carp, byproducts and waste, hydrolyse, proteins, amino acid composition

## Abstract

Collagen isolated from byproducts of common carp was hydrolyzed with alcalase enzyme to obtain peptide fractions. The resulting >30 kDa (PF1), 10–30 kDa (PF2), 3–10 kDa (PF3) and <1 kDa (PF4) fractions were studied for their antioxidant and functional properties. All peptide fractions illustrated antioxidant activity at different concentrations (1, 5, and 10 mg/mL). Although PF4 indicated the highest DPPH radical-scavenging activity (87%) at a concentration of 1 mg/mL, the highest reducing power (0.34) and hydroxyl radical scavenging activity (95.4%) were also observed in PF4 at a concentration of 10 mg/mL. The solubility of the peptide fractions was influenced by pH. The lowest solubility of the peptide fractions was observed at pH 4. The highest emulsifying activity index (EAI) was observed for PF4 (121.1 m^2^/g), followed by PF3 (99.6 m^2^/g), PF2 (89.5 m^2^/g) and PF1 (78.2 m^2^/g). In contrast to what has been found in the case of EAI, the emulsion stability of the peptide fractions decreased at lower molecular weight, which ranged from 24.4 to 31.6 min. Furthermore, it was revealed that PF1 had the highest foam capacity (87.4%) and foam stability (28.4 min), followed by PF2 and PF3. Overall, the findings suggest that peptide fractions isolated from byproducts of common carp are a promising source of natural antioxidants for application in functional food and pharmaceutical products.

## 1. Introduction

Fish is considered highly perishable food, i.e., starts to spoil immediately after being caught. It is necessary to employ different processing methods, such as filleting, packing, salting, or smoking, to improve fish shelf life [1]. These methods usually produce high quantities of byproducts, i.e., between 30 and 70% of total fish mass, representing an interesting source of micro- and macronutrients that, in most cases, are not properly exploited [2]. Considering the growing demand for fish products on a global level (179 million tons in 2018, which is expected to increase to 204 million tons in 2030 [3]), it is necessary to find new forms to valorize fish byproducts and avoid the environmental impact related to the inappropriate disposal of these residues.

Filleting is the main fish processing step, generating up to 70−75% of solid byproducts such as bones, skins, heads, scales, skeletons, tails, viscera, etc. These byproducts represent a valuable source of collagen and gelatin with a large quantity of high-quality proteins [4,5]. Collagen is a connective protein-based tissue which contains hydroxyproline, proline and glycine as major amino acids, and presents a huge potential to produce bioactive peptides through hydrolysis [6]. Fish byproducts have proved to be an interesting alternative for obtaining collagen in comparison with traditional sources, i.e., mainly skins and bones of pigs or cows, since fish-derived collagen is not considered a potential spreader of diseases (such as bovine spongiform encephalopathy or foot and mouth disease). Additionally, its use is not associated with religious controversies, like cow-derived collagen in India or pig-derived collagen among Muslim and Jewish communities [7].

Enzymatic hydrolysis of proteins yields bioactive peptides with several interesting characteristics, such as antioxidant, antimicrobial, antihypertensive or antithrombotic properties, as well as a high content of essential amino acids [8,9]. Enzymatic treatment is also preferred since it avoids toxic chemicals and residual organic solvents, which is especially important in food and pharmaceutical industries. Some commercial enzymes commonly used in the enzymatic hydrolysis of fish proteins are alcalase, papain, pepsin, trypsin, and pancreatin. Specifically, alcalase has proven to have an excellent capacity to generate bioactive peptides from different types of fish byproducts, such as yellowfin tuna heads [10], aquaculture turbot wastes [11] or salmon skin [12]. Previous researchers have reported that alcalase was the most effective among several proteases in the hydrolyzation gelatin from skipjack *Katsuwonus pelamis* scales; the resulting peptides displayed strong antioxidant activities [13].

Aerobic life entails the generation of reactive oxygen species (ROS) which participate in some cell signal processes but which also induce deleterious effects on different macromolecules, including DNA. The oxidative stress produced by ROS can trigger diseases like Alzheimer’s, Parkinson’s, diabetes, cardiovascular disorders and cancer [14]. Fish-derived bioactive peptides represent a valuable source of natural antioxidants capable of scavenging ROS and reducing cellular damage. Furthermore, in vivo research has also demonstrated that bioactive peptides with high antioxidant capacities also possess strong ACE-inhibitory, antimicrobial, and anti-inflammatory activities [15]. This is especially relevant since some synthetic antioxidants are suspected of causing a potential risk to human health in high doses [15], thus making it interesting to find bio-based antioxidant alternatives.

Common carp (*Cyprinus carpio*) is considered one of the most relevant species of freshwater fish. It is usually reared in polyculture, semi-intensive systems along with other species belonging to the cyprinidae family such as silver carp (*Hypophthalmichthys molitrix*), grass carp (*Ctenopharyngodon Idella*) and bighead carp (*Aristichthys nobilis*). Although common carp is native to Central Asia and Eastern Europe, this species has spread to more than 120 countries where it is currently cultured and consumed [16]. Freshwater fish production depends largely on carp species, with common carp being the fourth most-produced fish in aquaculture globally [17]. Several studies have assessed the antioxidant capacity of protein hydrolysates from common carp residues and roe [18]. As far as we know, no studies to date have focused on the antioxidant and techno-functional properties of bioactive peptides derived from collagen hydrolysate of common carp byproducts. Lastly, this paper aims to find an alternative way to valorize and use the fish byproduct while also managing waste generation. Therefore, the antioxidant and functional properties of four peptide fractions derived from the collagen hydrolysate of common carp were investigated.

## 2. Materials and Methods

### 2.1. The Extraction of Collagen from Common Carp Fish Byproduct

The mixed byproducts used in this study contained heads, skins, and skeletons of common carp fish; they were obtained from the filleting process line of Khazar shilat Co., Gorgan, Iran. The isolation of collagen was carried out based on the method presented by Zamorano-Apodaca (2020) [5], with minor modifications. Firstly, noncollagenous proteins were eliminated by soaking the byproduct in 0.1 M NaOH using a solid material to alkaline solution ratio of 1:10 (*w*/*v*) for 8 h. Also, the extracted collagen was bleached with 0.5% NaClO with 1:4 (*w*/*v*) for 30 min. The collagen was extracted with 0.5 M acetic acid solution for 24 h with a solid/solution ratio of 1:10 (*w*/*v*) at 25 °C. The mixture was then centrifuged (MPW-260, Warsaw, Poland) at 10,000× *g* for 20 min before dialysis was carried out using 20 volumes against 0.1 M acetic acid. The retentate was freeze-dried (Multi-manifold, Tamil Nadu, India) and stored at −80 °C until further analysis.

### 2.2. Preparation of Collagen Hydrolysate

The obtained collagen was hydrolyzed according to the method of Sun et al. (2022) [19] with some modifications. Collagen powder was dissolved in deionized water at a concentration of 6.67% (*w*/*v*) and adjusted to pH 8.0 with NaOH (1 M). Then, the solution was hydrolyzed with 2% alcalase (*v*/*v*) from *Bacillus licheniformis* (≥2.4 U/g, Sigma Aldrich, St. Louis, MO, USA) at 55 °C for 3 h. For enzyme inactivation, the solution was kept at −20 °C for 10 min.

The mixture was centrifuged at 10,000× *g* at 4 °C for 15 min, and the supernatant was obtained as the hydrolysate. The hydrolysis degree was measured by determining the free amino groups (NH_3_) via the trinitrobenzyl sulfonic acid (TNBS) technique [5].

### 2.3. Isolation of Peptide Fractions

The four peptide fractions were separated by sequential ultrafiltration (Millipore Co. Waltham, MA, USA) with molecular weight cut-offs of 30, 10, and 3 kDa. Thus, four fractions (retentate) were obtained >30 kDa (PF1), 10–30 kDa (PF2), 3–10 kDa (PF3) and <3 kDa (PF4), and immediately freeze dried. The percent yields of the fractions were calculated as the ratio of peptide weight of each fraction to peptide weight of the hydrolysate.

### 2.4. Amino Acid Analysis

The amino acid composition was estimated according to the method described by Hadidi et al. (2021) [20] by high performance liquid chromatography (HPLC) system (Shimadzu, Tokyo, Japan) with a fluorescence detector (RF-20AXS, Shimadzu, Tokyo, Japan). First, 50 mg of samples were hydrolyzed by 2 mL of 6 N HCl at 110 °C for 24 h. Next, 10 μL of samples was added to 20 μL reagent (AccQ-fluor derivatization) and mixed at 55 °C for 10 min. A mixture of acetonitrile (60% *v*/*v*) and sodium acetate buffer (pH 4.9) was used as an eluent.

### 2.5. Antioxidant Activities

#### 2.5.1. DPPH Radical-Scavenging Activity

The method of Haghani et al. (2021) [21] was used to determine DPPH free radical scavenging activity of the peptide fractions. First, 50 μL of the peptide fractions at 1, 5, and 10 mg/mL (in MilliQ water) was mixed with 500 μL of 0.1 mM DPPH in 95% (1:1, *v*/*v*) ethanol and incubated with shaking for 30 min at room temperature. Then, the absorbance was read at 517 nm using a UV-Visible spectrophotometer (DS5, Edinburgh Instruments, Livingston, UK). A control (without sample) and blank (95% ethanol) were included. Ascorbic acid was included as the positive control. The DPPH radical scavenging activity was calculated as follows:

DPPH radical scavenging activity (%) = (A_0_ − A_1_)/(A_0_) × 100%

where A_0_, A_1_ is the absorbance of the control at 517 nm and the absorbance in the presence of sample, respectively. 

#### 2.5.2. Hydroxyl Radical Scavenging Activity

Hydroxyl radical scavenging activity was measured colorimetrically by a UV-Visible spectrophotometer at 536 nm based on the method of Zamorano-Apodaca et al. (2020) [5]. First, 0.2 mL of the peptide fractions at concentrations of 1, 5, and 10 mg/mL (in distilled water) was mixed with 0.9 mL of sodium phosphate buffer (pH 7.4), 0.1 mL of 10 mM ethylenediaminetetraacetic acid (EDTA), 0.1 mL of FeSO_4_ (10 mM), and 0.5 mL of α-deoxyribose (10 mM). Next, 0.2 mL of H_2_O_2_ (10 mM) was added to start the reaction. After incubation in darkness at 37 °C for 1 h, 1.0 mL of trichloroacetic acid (2.8%) was added to stop the reaction. Subsequently, 1.0 mL of 1.0% 2-thiobarbituric acid (TBA) was added, mixed, and kept in a boiling water bath for 20 min to develop color. After cooling, the absorbance was determined to be 532 nm. Distilled water was used as the blank. Ascorbic acid was used as the positive control. The hydroxyl radical scavenging activity was calculated using the following equation:

Hydroxyl radical scavenging activity (%) = (A_0_ − A_1_)/(A_0_) × 100%

where A_0_, A_1_ is the absorbance of the control at 532 nm and the absorbance in the presence of sample, respectively. 

#### 2.5.3. Reducing Power

First, 100 μg of peptide fractions (at concentration of 1, 5, and 10 mg/mL) were added to 100 μL 0.2 M phosphate buffer (pH 6.6) and 100 μL 1% potassium ferricyanide and incubated at 50 °C for 20 min. Plates were incubated for another 10 min at 50 °C after the addition of 100 μL 10% tricholoacetic acid and 20 μL 0.1% ferric chloride. Finally, absorbance was read at 700 nm [5].

### 2.6. Functional Properties

#### 2.6.1. Solubility

The solubility of the peptide fractions was determined according to a previously reported method with some modifications [22]. Briefly, one gram of each peptide fraction was dispersed in 100 mL phosphate buffer (0.01 M) at pH 4.0, 7.0, and 10.0. 2 M NaOH or 2 M HCl were used for adjustment of the pH. The mixture was kept in orbital shaking for 1 h at 25 °C. The supernatant was separated after centrifuging at 10,000× *g* for 15 min. The concentration of protein was then calculated based on the protein standard curve obtained using a Pierce™ BCA Protein Assay Kit (Thermo Fisher Scientific, Waltham, MA, USA). The extent of solubility was calculated by the division of protein content (mg) in the supernatant and the total weight of sample (1000 mg).

#### 2.6.2. Emulsifying Properties

The emulsion activity index (EAI) and stability index (ESI) of the four peptide fractions were determined by modifying the method defined by Pearce and Kinsella (1978) [23]. A protein suspension with a concentration of 10 mg/mL was prepared in distilled water at pH 7. The solution (8 mL) was mixed and homogenized with sunflower oil (2 mL) using a homogenizer (SBS-MR-2500, Steinberg systems, Zielona Gora, Poland) at 20,000 rpm for 1 min. After that, the emulsion (25 µL) was taken from the bottom of the centrifuge tube and then added to SDS solution (2.5 mL, 0.1% *w*/*v*). The absorbance of the mixture was determined at 500 nm using a UV–Vis spectrophotometer (DS5, Edinburgh Instruments, Livingston, UK) at 0 (A_0_) and 10 min (A_10_), whereby 0.1% SDS was considered as a blank. EAI and ESI were calculated as follows:

$$\mathrm{EAI}\;\left(m^{2}/g\right)=\frac{2\times 2.303\times {\;A}_{0}\times \mathrm{DF}}{c\;\times \varphi \;\times 1000}$$

$$\mathrm{ES}\;\left(\min\right)=\frac{A_{0}}{A_{10}-{\;A}_{0}}\times 10$$

where A_0_ and A_10_ are absorbance at 0 and 10 min, respectively; DF represents the dilution factor; and c and φ are the concentration of peptide fraction (g/mL) and the oil faction (*v*/*v*), respectively.

#### 2.6.3. Foam Properties

The foaming properties of the peptide fraction were measured according to the method described in a previous report [24]. First, foam was fabricated by stirring the sample mixture (10 mL, 1 mg/mL) for 2 min at 15,000 RPM. Foaming capacity (FC) and foam stability (FS) were calculated as follows:

$$\mathrm{FC}\;\left(\%\right)=\frac{V_{0}-{\;V}_{b}}{V_{b}\;}\times 100$$

$$\mathrm{FS}\;\left(\%\right)=\frac{V_{30}-{\;V}_{0}}{V_{0}\;}\times 100$$

where V_b_ represents the volume of the suspension before homogenization, and V_0_ and V_30_ are the volume of the suspension after homogenization at 0 min and 30 min, respectively.

### 2.7. Statistical Analysis

Experimental data were analyzed with ANOVA (one-way analysis of variance). The significant difference among the mean values was examined by Duncan’s test (*p* ≤ 0.05) with SPSS 23.0 software (SPSS Inc., Chicago, IL, USA).

## 3. Results and Discussion

### 3.1. Amino Acid Composition of the Extracted Collagen and Their Peptide Fractions

The yields of peptide fractions for PF1, PF2, PF3, and PF4 were 21.4, 26.6, 18 and 31.7%, respectively. The antioxidant and functional properties of peptides are directly related to their amino acid composition, as well as their structure and hydrophobic properties [25]. The amino acid composition of all peptide fractions and extracted collagen is shown in Table 1. Both collagen and peptide fractions showed different types of amino acids that were capable of contributing to antioxidant activity: hydrophobic amino acids (isoleucine, leucine, methionine, phenylalanine, tyrosine, threonine, valine, alanine, glycine, proline and serine), amino acids with the capacity of chelating pro-oxidative transition metals (histidine, aspartic/asparagine and glutamic/glutamine) and amino acids capable of donating electron/hydrogen (glutamic/glutamine and methionine) [26]. Arginine, lysine and histidine, i.e., amino acids with positive charge and associated with antimicrobial activity, are also present in all fractions [27]. The most abundant amino acids identified in collagen and PF1-PF4 were glycine (28% in collagen, 23% in PF1-PF3 and 15% in PF4), proline (13% in collagen, 9% in PF1-PF3 and 3.5% in PF4), glutamic/glutamine (10% in collagen and PF1-PF2, 11% in PF3 and 7% in PF4), arginine (9% in collagen and PF2, 11% in PF1, 10% in PF3 and 6% in PF4), alanine (8% in collagen, 10% in PF1-PF2, 9% in PF3 and 11% in PF4) and aspartic/asparagine (5% in collagen and PF1-PF2, 6% in PF3 and 17% in PF4). All these amino acids have properties that allow them to increase the antioxidant activity of peptides [25], except for arginine, which is positively charged and contributes to antimicrobial activity [26]. It is important to note that even though the quantity of amino acids in PF4 was considerably lower than the values obtained for other fractions, the histidine and aspartic/asparagine content in PF4 were significantly higher (10% and 17%, respectively, in contrast to 3–8% and 5–6% in all other cases). Amino and carboxylic groups from aspartic/asparagine, as well as imidazole ring from histidine, have proved to possess a strong pro-oxidative transition metal-chelating capacity [15,26]. This fact could explain the higher antioxidant activity observed by PF4 in comparison to all other fractions (Table 2).

### 3.2. Antioxidant Activity of Peptide Fractions

#### 3.2.1. DPPH Radical Scavenging Activity

DPPH˙ displays a strong absorption band at 517 nm as a stable radical, which vanishes in the presence of free radical scavengers, since the DPPH free electron is paired off [15]. The capacity of peptide fractions to scavenge DPPH˙ radicals is shown in Table 2; notably, all peptide fractions had a DPPH radical scavenging activity superior to 60%. The highest scavenging activity (87%) was for PF4 (<3 kDa) at 1 mg/mL, i.e., very close to the value displayed by the positive control at the same concentration (88%). These results were superior to those reported by Chi et al. using Spanish Mackerel skin (scavenging activity below 30% for peptide fractions superior to 30 kDa) [27] and were in agreement with those obtained by Zamorano-Apodaca et al. with byproducts of different fish species (maximum DPPH˙ scavenging activity of 81% for <3 kDa fraction at 5 mg/mL) [5]. The DPPH radical scavenging activity was dependent on concentration (*p* < 0.05) in all peptide fractions. Only in the case of PF4, the radical activity increased when the concentration decreased; the opposite trend was observed in all other fractions. This fact could be explained by considering that in the case of low molecular weight fractions, the exposure of certain amino acids, such as alanine, glycine, or glutamic acid, could be favored, thereby resulting in higher interaction between peptides and lipid substances and promoting the stability of free radicals [28]. In general, the good results obtained could be associated with the presence of electron/hydrogen-donor peptides in the collagen hydrolysates, which interrupt radical chain reactions thanks to their capacity to react with free radicals [15].

#### 3.2.2. Hydroxyl Radical Scavenging Activity

Hydroxyl radicals (OH˙) are among of the most reactive oxygen species; they pose a potential risk to several cellular structures [29]. In our work, the hydroxyl radical scavenging activity of all peptide fractions assayed was superior to 60%, except for PF1 (>30 kDa) at 1 mg/mL, which showed an activity of 53% (Table 2). The maximum OH˙ scavenging activity obtained was 95% for PF4 (<3 kDa) at 10 mg/mL, which was higher than the maximum reported by Zamorano-Apodaca et al. (89% at 15 mg/mL for <3 kDa fraction) [5]. In all fractions, it was possible to notice a dependency between OH˙ scavenging activity and concentration (*p* < 0.05), in such a manner that scavenging activity increased when the concentration was higher. This direct relationship between OH˙ scavenging activity and concentration was also observed in PF4 (<3 kDa), indicating that a higher exposure of small peptide fractions has no negative impact in the case of OH˙ deactivation. The good results obtained in most of the fractions could be related to the activity of electron/hydrogen-donor peptides, as well as the presence of hydrophobic amino acids in the composition of these peptides (Table 1). Some amino acids detected also have the ability to chelate pro-oxidative transition metals, thus favoring the reduction and deactivation of OH˙ free radicals [28]. All these facts could explain the dependency between OH˙ scavenging activity and concentration in all fractions.

#### 3.2.3. Reducing Power

Reducing power assays are commonly employed to measure the capacity to donate electron/hydrogen from natural antioxidants to free radicals [15]. The results collected in Table 2 show that there is a dependency between concentration and reduction power for all fractions (*p* < 0.05), i.e., higher concentrations imply higher reducing power. Lower-molecular-weight fractions show better reduction power than others, and the best result reported (reducing power of 0.34) was achieved by PF4 (<3 kDa) at 10 mg/mL, similar to the results of Zamorano et al. under the same conditions (0.345 at 10 mg/mL for <3 kDa fraction) [5]. It is important to note that the highest reducing power obtained was only about 25% of the result displayed by ascorbic acid at 10 mg/mL. The superior performance of ascorbic acid in comparison to bioactive peptide fractions has been described by other authors [5,15]; it could be explained by considering the lactone structure of this organic acid, which allows the donation of electrons from the unsaturated ring to free radicals [29]. This electron-donation process could be more efficient than donation from electron/hydrogen donor peptides, thus explaining the difference between the results obtained by peptide fractions and the positive control.

### 3.3. Functional Properties of Peptide Fractions

#### 3.3.1. Solubility

**Figure 1:**
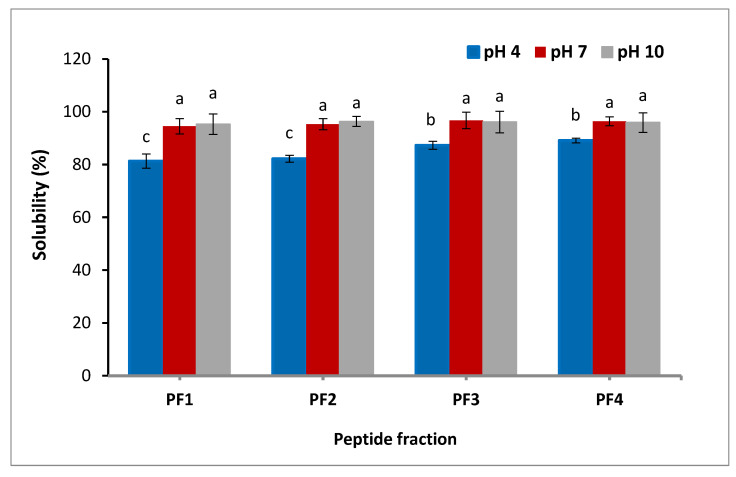
Solubility of the isolated peptide fractions at pH 4.0, 7.0, and 10.0. Results are illustrated as means ± SD and values within each column with the different letters are significantly different (*p* < 0.05).

Solubility is one of the most critical factors in the development of proteins and their hydrolysates. It can influence other functionalities of proteins, like foaming and emulsifying properties [5]. As presented in Figure 1, the solubility of the peptide fractions was found to be influenced by pH; the lowest solubility of the peptide fractions was observed at pH 4. The results confirmed that with an increment of pH from 4 to 7, a much higher pH of the peptide fractions was attained. No significant change was exhibited in the solubility as the pH further increased. At pH 4, the solubilities of PF1, PF2, PF3, and PF4 were 81.3, 82.2, 87.3, and 89.1%, respectively, indicating that the solubility increased at lower size, probably due to the fact that small molecules are more polar. Due to the electrostatic interactions of the amino acids and their ability to form hydrogen bonds with water, the solubility of peptide fractions was suitable [30]. The lowest solubility of salmon byproduct hydrolysates was detected at pH 4 [31]. Differences in the solubility of the peptide fractions might be ascribed to both peptide net charge, which increases as pH moves away from the isoelectric point, and surface hydrophobicity, which enhances aggregation by hydrophobic contact [32]. These findings are in agreement with the results of the skin gelatin hydrolysates study, which reported that the solubility of the gelatin hydrolysates derived from sole fish skin decreased at lower pH [33].

#### 3.3.2. Emulsifying Properties

**Figure 2:**
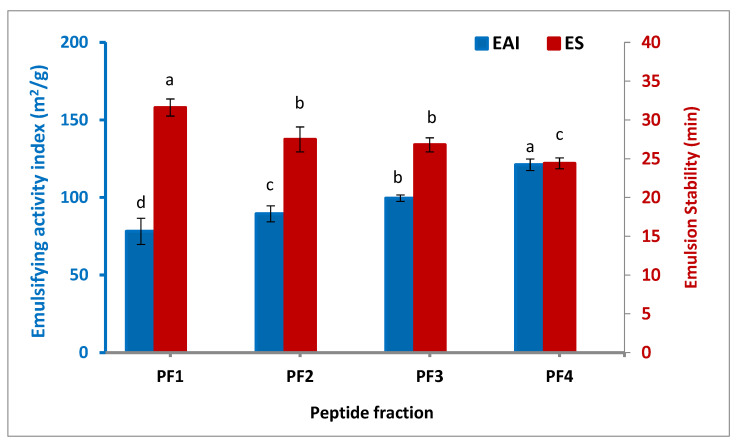
Emulsifying activity index and stability of the isolated peptide fractions. Results are illustrated as means ± SD and values within each variable with the different letters are significantly different (*p* < 0.05).

Figure 2 illustrates the emulsifying activity index (EAI) and emulsion stability (ES) of the isolated peptide fractions. The highest EAI was observed for PF4 (121.1 m^2^/g), followed by PF3 (99.6 m^2^/g), PF2 (89.5 m^2^/g), and PF1 (78.2 m^2^/g), denoting a significant increase in EAI when the molecular weight of the peptide fraction was lower (*p* < 0.05). The same trend was also found by Zamorano-Apodaca et al. [5], who reported that when the molecular weight of the peptide fractions decreased, the EAI and superficial hydrophobicity increased by the higher generation of small fat globules, allowing molecular rearrangement and unfolding to occur at the emulsion interface, and hence, the subsequent diffusion at the interface between oil and water. In contrast to what was found in the case of EAI, the ES of the peptide fractions decreased at lower molecular weight, which varied from 24.4 to 31.6 min. In terms of minimizing interfacial tension, smaller peptides are less effective, but this is not enough to improve their emulsion stability because of the lack of viscosity and reorientation at the interface. As a result, they quickly disrupt the emulsion process. Similar studies have observed that emulsion stability is improved with increasing the molecular weight of peptides [34,35].

#### 3.3.3. Foaming Properties

**Figure 3:**
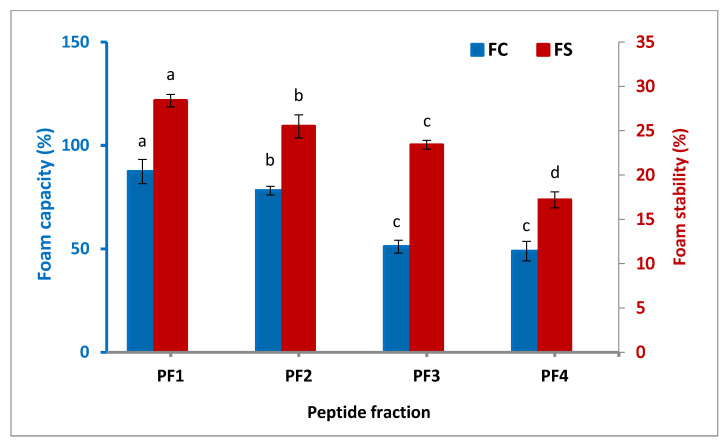
Foam capacity and stability of the isolated peptide fractions. Results are illustrated as means ± SD and values within each variable with the different letters are significantly different (*p* < 0.05).

The foam capacity (FC) and foam stability (FS) of the isolated peptide fractions as two major parameters for the foaming characteristic of proteins and peptides are indicated in Figure 3. It was found that PF1 had the highest FC (87.4%) and FS (28.4 min), followed by PF2 and PF3. The FC and FS of the isolated peptide fractions were enhanced significantly (*p* < 0.05) with the increasing molecular weight of the fractions. Peptide fractions with high molecular weights show the needed force to form foam due to their ability to reorganize their structure at the air–water interface [36]. Three elements influence foam formation: the transportation, penetration, and restructuring of molecules at the water–air interface. A protein with a good foam forming property must be capable of migrating quickly to the water–air interface, unfolding, and reorganizing there [37]. A study on the functional properties of different peptide fractions extracted from a byproduct of several fish species by Zamorano-Apodaca et al. (2020) also showed peptide fractions; the study reported that molecular weights of more than 30 kDa possessed a stronger FC and FS than those with lower molecular weights [5].

## 4. Conclusions

In conclusion, this study revealed that four peptide fractions derived from the collagen hydrolysis of common carp fish byproducts possessed antioxidant and functional properties. The highest antioxidant activity (DPPH and hydroxyl radical scavenging activities and reducing power) was for the low molecular weight peptide fraction (PF4, <3 kDa). In addition, the solubility of the peptide fractions was affected by pH. The PF1 (>30 kDa) had the greatest foam capacity, foam stability, and emulsifying activity index, but the lowest emulsion stability. As a result, peptide fractions of collagen derived from common crab fish byproducts produced by larger fish processing companies might be a viable option as a functional ingredient for several industries.

## Figures and Tables

**Table 1:** Amino acid composition (expressed as % of total amino acids) of extracted collagen and peptide fractions.

Amino Acid	Collagen	PF1 (>30 kDa)	PF2 (10–30 kDa)	PF3 (3–10 kDa)	PF4 (<3 kDa)
**Essential amino acid**					
Histidine (His)	0.97	2.62	6.58	7.41	10.67
Isoleucine (Ile)	0.85	1.45	1.49	1.17	1.46
Leucine (Leu)	1.91	1.88	1.67	1.34	3.35
Lysine (Lys)	3.67	4.19	4.48	4.19	5.02
Methionine (Met)	0.48	0.74	0.83	0.80	ND
Phenylalanine (Phe)	0.58	1.08	1.20	0.95	1.26
Tyrosine (Tyr)	0.43	0.49	0.41	0.41	0.94
Threonine (Thr)	5.02	4.85	3.86	2.89	5.54
Valine (Val)	1.25	2.45	2.39	2.01	1.57
**Non-essential amino acid**					
Alanine (Ala)	8.35	10.20	10.42	9.42	11.61
Aspartic/asparagine (Asp)	4.62	5.07	4.81	5.55	17.47
Arginine (Arg)	9.31	11.51	9.51	10.13	6.49
Glycine (Gly)	27.81	23.25	23.39	23.70	15.06
Glutamic/glutamine (Glu)	10.02	10.67	10.64	11.45	7.22
Proline (Pro)	12.89	9.53	8.99	9.05	3.66
Hydroxyproline (Hyp)	9.30	7.65	6.70	6.72	5.75
Serine (Ser)	2.55	2.37	2.64	2.81	2.93

**Table 2:** Antioxidant activity of the isolated peptide fractions.

Peptide Fraction	Concentration (mg/mL)	DPPH Radical Scavenging Activity (%)	Hydroxyl Radical Scavenging Activity (%)	Reducing Power (OD at 700 nm)
PF1 (>30 kDa)	1	61.83 ± 1.35 ^h^	53.70 ± 1.35 ^k^	0.07 ± 0.01 ^j^
	5	65.76 ± 1.43 ^g^	61.06 ± 0.86 ^j^	0.09 ± 0.01 ^h,i^
	10	71.33 ± 0.76 ^f^	66.36 ± 1.01 ^i^	0.12 ± 0.01 ^g,h^
PF2 (10–30 kDa)	1	65.93 ± 0.93 ^g^	66.80 ± 1.44 ^i^	0.09 ± 0.01 ^h,i^
	5	70.93 ± 0.97 ^f^	71.13 ± 1.60 ^h^	0.148 ± 0.004 ^g^
	10	76.53 ± 1.25 ^e^	77.26 ± 0.87 ^g^	0.184 ± 0.010 ^f^
PF3 (3–10 kDa)	1	72.70 ± 1.31 ^f^	76.96 ± 1.79 ^g^	0.115 ± 0.007 ^g,h^
	5	77.60 ± 1.13 ^e^	86.86 ± 1.25 ^f^	0.18 ± 0.01 ^f^
	10	84.46 ± 1.09 ^c^	91.46 ± 1.02 ^d^	0.24 ± 0.01 ^e^
PF4 (<3 kDa)	1	87.06 ± 1.40 ^b^	88.76 ± 0.76 ^e^	0.138 ± 0.003 ^g^
	5	82.23 ± 0.90 ^d^	91.80 ± 0.70 ^d^	0.25 ± 0.01 ^e^
	10	76.20 ± 1.15 ^e^	95.46 ± 0.56 ^b,c^	0.34 ± 0.01 ^d^
Ascorbic acid	1	88.73 ± 1.36 ^b^	94.10 ± 0.52 ^c^	0.64 ± 0.01 ^c^
	5	94.36 ± 0.98 ^a^	96.83 ± 0.61 ^a,b^	0.82 ± 0.03 ^b^
	10	95.46 ± 1.20 ^a^	98.26 ± 0.35 ^a^	1.42 ± 0.06 ^a^

Results are illustrated as means ± SD and values within each column with the different letters are significantly different (*p* < 0.05).

## Data Availability

The data presented in this study are available in the article.
